# Chronic inflammatory demyelinating polyradiculoneuropathy relapse after mexiletine withdrawal in a patient with concomitant myotonia congenita: A case report on a potential treatment option

**DOI:** 10.1097/MD.0000000000021117

**Published:** 2020-07-10

**Authors:** Simona Portaro, Fiammetta Biasini, Placido Bramanti, Antonino Naro, Rocco Salvatore Calabrò

**Affiliations:** IRCCS Centro Neurolesi Bonino Pulejo, Messina, Italy.

**Keywords:** chronic inflammatory demyelinating polyradiculoneuropathy, mexiletine, myotonia congenita

## Abstract

**Introduction:**

we report on the first case of a woman affected by chronic inflammatory demyelinating polyradiculoneuropathy (CIDP) and recessive myotonia congenita (MC), treated with mexiletine. We aimed at describing the possible role of mexiletine in CIDP management.

**Patient Concerns:**

A 44-year-old female affected by CIDP and MC, gained beneficial effects for CIDP symptoms (muscle weakness, cramps, and fatigue) and relapses, after mexiletine intake (200 mg twice a day). The patient presented with detrimental effects after mexiletine drop out, with a worsening of CIDP symptoms.

**Interventions:**

The patient reported a nearly complete remission of muscle stiffness and weakness up to 3 years since mexiletine intake. Then, she developed an allergic reaction with glottis edema, maybe related to mexiletine intake, as per emergency room doctors’ evaluation, who suggested withdrawing the drug.

**Outcomes:**

The patient significantly worsened after the medication drop out concerning both CIDP and MC symptoms.

**Conclusion:**

This is the first report on the association of CIDP and MC in the same patient. Such diseases may share some clinical symptoms related to a persistent sodium currents increase, which maybe due either to the over-expression of sodium channels following axonal damage due to demyelination or to the chloride channel genes mutations. This is the possible reason why mexiletine maybe promising to treat CIDP symptoms.

## 1 Introduction

Chronic inflammatory demyelinating polyradiculoneuropathy (CIDP) is an acquired immune mediated demyelinating disease of the peripheral nervous system (PNS), causing progressive loss of motor and sensory functions.^[1]^ CIDP belongs to the big family of the peripheral demyelinating diseases, which are a group of disorders damaging PNS axons and glial cells.^[2]^ The incidence of such peripheral demyelinating diseases is variable.^[3–5]^ CIDP pathogenesis is likely to be immune mediated.^[6]^ CIDP onset is insidious.^[7–10]^ Its clinical course is slowly progressive (over at least 2 months), with relapses, and includes symmetric weakness of both muscles around the hip and shoulder, as well as of the hands and feet (both proximal and distal muscles), and sensory disturbances causing incoordination, numbness, tingling, or prickling sensations.^[7–10]^ Other CIDP symptoms may include fatigue, burning sensation, pain, clumsiness, difficulty in swallowing, and double vision. Deep tendon reflexes are reduced or absent. Walking may be involved when responses to various sensory stimuli are seriously impaired.^[7–10]^ The immune system firstly attacks the myelin sheaths of the PNS, inducing the typical segmental demyelination and then axonal degeneration.^[9]^ This gets slower nerve conduction velocity, reduces sensory and motor responses amplitude, and provokes conduction blocks at the neurophysiological studies.^[9]^ To date, oral glucocorticoids are considered the first line treatment,^[11]^ together with plasmapheresis and intravenous human immune globulin (IVHIG).^[1,12]^

Myotonia congenita (MC) belongs to the nondystrophic myotonias, which are a group of genetically and clinically heterogeneous ion channel muscular disorders, typically characterized by delayed muscle relaxation after voluntary contraction, stiffness, muscle hypertrophy, and cramping.^[13]^ MC is caused by mutations in the skeletal muscle chloride channel gene (*CLCN1*). There are 2 forms, the autosomal dominant (Thomsen MC) and the autosomal recessive (Becker MC) MC. The latter is associated to transient weakness, with recovery often induced by repetitive exercises.^[13]^ Mexiletine is the first choice treatment to improve myotonia-induced symptoms.

Despite MC and CIDP are distinct clinical entities, they may share a common pathophysiological issue, that is, the channelopathy (the former is genetic and involves the muscle tissue, the latter is acquired and affects the peripheral nerve). Thus, a possible association between these 2 clinical conditions maybe not necessarily casual.

Herein, we report on the first case of a woman affected by CIDP and MC, treated with mexiletine. The patient got detrimental effects on CIDP symptoms after mexiletine drop out. The patient provided her informed consent for publication of the case

## 2 Case report

In 2002, a 44-year-old female began to complain of symmetric proximal and distal weakness with difficulty in walking, mild sensory dysfunction with distal limb not-disabling paresthesia, and absent tendon reflexes in all extremities, which all gradually worsened over 2 months. She was then hospitalized elsewhere, and she underwent several investigations, including:

(1) electroneuromyography (EMG) showing peripheral nerve demyelination: that is, conduction blocks, a reduction of nerve conduction velocities (NCV) <80% of lower limit of normal, with a discrete variability among nerves, dispersed compound muscle action potentials (CMAP), and prolonged distal latencies, as well as F-waves presence;
(2) needle EMG showing no abnormal spontaneous activity, prolonged motor unit action potentials, and a reduced interference pattern), and
(3) lumbar puncture, disclosing increased protein level (67 mg/dL) with a normal cell count (<5 mononuclear cells) in the cerebrospinal fluid. She was; therefore, diagnosed with CIDP, according to the EFNS/PNS diagnostic criteria for CIDP.^[14]^

She was treated with prednisone 100 mg/d p.o., which was withdrawn after the clinical improvement (about 2 weeks later). Within 1 month, she was at the level she had been before CIDP onset, but a residual mild numbness of fingertips and muscle stiffness were appreciable. There were no limitations in her daily life activities. The EMG pattern consisted of an improvement in CMAP amplitude, reduced distal latencies, improved NCV and F-wave latency, and conduction block disappearance. However, she was prescribed with methotrexate 25 mg per week, followed by folin administration the day after the injection. Over the years, she had 5 relapses (from 2002 to 2015), each of them characterized by mild distal limb paresthesia progressing to forearms and upper legs, diffuse weakness of arm and leg muscles, until she was not able to walk without assistance. Each relapse was treated with high-dose iv corticosteroids (methylprednisolone 1 g iv/d for 5 days, then tapered with additional 1 g iv/d a week for 4 weeks), and the methotrexate was supplemented with monthly iv pulse of cyclophosphamide for 6 months, with clinical beneficial effects each time. Indeed, she was at the level she had been before the relapse, within 10 days from iv corticosteroids, with only residual mild lower limbs muscle weakness and stiffness. Notably, since 2008, the patient began to use myorelaxant as needed to face the increasing muscle stiffness. No data on other EMG investigations were available.

In 2015, her daughter was diagnosed with recessive MC due to the Gly190Ser mutation in the *CLCN1* gene at our neuromuscular specialized center. Thus, the patient took part to a familial screening for MC, being provided with a neurophysiological examination following the Fournier guidelines for ion channel disorders. The exam confirmed a chloride muscle channelopathy, as in her daughter. The EMG pattern showed a reduced CMAP amplitude and NCV, and an increase in F-wave latency. The Fournier pattern II consisted of post-exercise myotonic potentials, a transient decline of CMAP amplitude after short exercise test after rest, and a reversion of the block of muscle excitability induced by repeating short exercise test. Unfortunately, the diagnosis was not supported by molecular analysis, since the patient refused to do it. In consideration of the residual symptoms (mild lower limbs muscle weakness and stiffness) and the probable MC diagnosis, the patient was then prescribed mexiletine (200 mg twice a day) and monitored by serial electrocardiograms to check the QT interval.

The patient reported a nearly complete remission of muscle stiffness and weakness up to 2018. Also, she reported a CIDP remission, complaining only of mild numbness of fingertips. She did not practice any other specific investigation. Because the patient felt such a clinical improvement, she decided to suspend on her own the methotrexate assumption after 2 months from the mexiletine intake.

Unfortunately, in 2018, she developed an allergic reaction with glottis edema, considered to be related to mexiletine by the emergency room doctors. Even though the cause of such allergy was neither tested nor confirmed, mexiletine was suspended. Some weeks later, the patient began to complain of walking problems with a progressive wide-based unsteady gait, mild distal lower limb paresthesia, muscle weakness, and fatigue. Such symptoms brought the patient to the Emergency Neurology Unit of our institute. On admission, the patient exhibited eyelid, lid-lag, handgrip and percussion myotonia, gait ataxia, difficulty in starting ambulation because of muscle stiffness, difficulty to walk on tiptoes and heels. Romberg sign was positive. There was a marked lower limb muscle weakness, more pronounced proximally. Deep tendon reflexes were decreased. She referred lower limb distal pain hypoesthesia and hypopallesthesia. EMG showed a pattern suggesting CIDP relapse (ie, worsening of CMAP amplitude, NCV, and F-wave latency, and the reappearance of conduction blocks); also, myotonic discharges were appreciable in all examined muscles. A diagnosis of CIDP relapse associated with MC was thus confirmed. The patient was provided with IVHIG at the dose of 400 mg/kg/d for 5 days, with a quick improvement in gait, motor, and sensitive symptoms. Neurological examination after IVHIG showed eyelid, lid-lag, handgrip and percussion myotonia, difficulty in starting ambulation because of muscle stiffness, but with normal muscle strength and sensibility at the 4 limbs. Deep tendon reflexes were still decreased, together with a slight lower limb distal pain hypoesthesia and hypopallesthesia. IVHIG administration was repeated every week for 4 months. Also, azathioprine was started at the dosage of 100 mg/d.

Actually, the patient complains only of the abovementioned MC-related symptoms. No CIDP relapses have yet occurred. The patient refused to reintroduce mexiletine because of the previous, even not confirmed, allergic reaction. The patient provided her written informed consent to study publication.

## 3 Discussion

CIDP and MC are rare disorders of the PNS. To date, there is no report on their association in the same patient. In our case, MC diagnosis followed CIDP diagnosis, and it was made in the context of a genetic familial screening for ion channel disorders. Despite MC and CIDP are distinct clinical entities, they may share a common pathophysiological issue, that is, the channelopathy (the former is genetic in origin and involves the muscle tissue, the latter is acquired and affects the peripheral nerve). The muscle chloride channelopathy represents the primary trait of MC. On the other hand, peripheral (and even central) nervous system channelopathies can be due to a demyelization process exposing channel structures at the level of nodes and paranodes.^[15–17]^ Despite the different origin of these channelopathies, a pathophysiological correlation may exist; for example, the nerve pathology could negatively affect muscle membrane physiology with a change in channel function or expression.^[18]^ Anyway, the channelopathy causes an abnormal gain of channel function (eg, myokymia, myotonia, and epilepsy) or an abnormal loss of channel function (eg, weakness or numbness), depending on whether the channelopathy leads to membrane hyper- or hypo-excitability.^[19]^ Thus, a possible association between these 2 clinical conditions maybe not necessarily casual.

This association is supported by some common positive (cramps, involuntary muscle twitching) and negative symptoms (muscle weakness) between such diseases. These symptoms can be caused by both the hyper- or hypo-excitability of the injured axons (secondary to the demyelization process) and by the muscle chloride channelopathy.^[20]^ Generally speaking, voltage-gated sodium channel (Na_v_) dysfunction causes a failure in inactivating and conducting sustained inward Na^+^ fluxes, with consequential skeletal muscle hyperexcitability, or weakness in presence of high levels of extracellular K^+^.^[21]^ Voltage gated potassium channel (K_v_) dysfunction is characterized by reduced K^+^ effluxes, thus prolonging the falling or repolarization phase of the action potential and inducing repetitive nerve firing (that causes excessive and unregulated neurotransmitter release at the neuromuscular junction).^[21]^ In addition, voltage gated chloride channel (Cl^−^) defects cause smaller Cl^−^ influxes that in turn implies that smaller influxes of Na^+^ are needed to reach the threshold for action potentials, enhancing muscle excitability.^[21]^ Lastly, voltage gated calcium channel (Ca^2+^) dysfunction induces muscle weakness and paralysis due to a persistent membrane depolarization that inactivates the Na_v_ channels.^[21]^

Channelopathy in MC is genetically defined, whereas channelopathy in CIDP is thought to be related to the damage induced by some antibodies involved in CIDP pathogenesis. In fact, physiologically, in order to maintain and organize the junctions at nodes of Ranvier, paranodes and juxtaparanodes there are the axoglial cell adhesion molecules,^[22]^ that is, nodal axonal neurofascin-186 (NF-186), NF-155, contactin-1, NrCAM bind gliomedin, which are involved in Na_v_ and K_v_ clustering, required for fast and saltatory action potential propagation.^[23,24]^ Autoantibodies against NF-186, NF-155, gliomedin, and contactin-1 have been described in demyelinating inflammatory neuropathies.^[25–29]^ Disruption of axon membrane induced by these antibodies leads to clustering defects of nodal-paranodal channels (Na^+^/K^+^ ATPase, Na^+^/Ca^2+^ exchangers and high density of Na_v_) and the exposure of the iuxtaparanodal K_v_, with either positive or negative symptoms as a consequence.

Therefore, changes in nodal-paranodal channel functions in CIDP can lead to an enhanced excitability or to an inexcitability (which causes positive and negative symptoms, respectively), depending on the prevailing channel dysfunction.^[30–32]^ This is what also occurs with the *CLCN1* mutation of MC.^[33]^

Moreover, in our case, the possible correlation between CIDP and MC is also suggested by the positive effect of mexiletine administration on both MC and CIDP symptomatology. Indeed, the administration of mexiletine resulted in a nearly complete remission of muscle stiffness, cramping, and weakness. Mexiletine (1-(2,6-dimethylphenoxy)-2-propanamine - C_11_H_17_NO) is a sodium channel blocker belonging to the class IB of antiarrhythmic agents, which is structurally related to the anesthetic lidocaine. It has been approved in Italy for the treatment of myotonia on 2006. The bioavailability is of 90%, and peak plasma concentrations occur after 2 to 4 hours. The mean drug half-life is of about 11 hours. The pharmacokinetics is preserved up to a creatinine clearance of 10 mL/min. It is metabolized by the liver, predominantly. Mexiletine prolongs the refractory period by delaying the recovery from inactivation of Na_v_. Specifically, Mexiletine targets the Na_v_ in a use-dependent and block voltage-dependent manner rather than blocking rest or tonically active Na_v_. Furthermore, it targets more downstream cell pathophysiology in the chloride channel.^[34]^ Therefore, Mexiletine is more active on muscle fibers subject to repeated discharges (like skeletal muscles in myotonia), thus reducing the delay in muscle relaxation and, eventually, muscle stiffness. Moreover, it is used for treating arrhythmias and chronic pain, consistently with its specific efficacy in blocking Na_v_ that continues to release a sustained current rather than fully inactivating.^[35–38]^

While the efficacy of mexiletine on muscle stiffness and cramping is intuitive and confirmed in different clinical trials,^[39,40]^ its effects on muscle weakness are less clear. We may speculate that mexiletine may have had challenged the abnormal nodal-paranodal channel activity (blockade of non-specific sodium channel) up to blocking glutamate-evoked activity in the dorsal horn of the spinal cord, beyond the well-known muscular effects.^[41]^ Such mechanism may confirm the pathophysiological correlation between CIDP and MC, and thus the drug maybe helpful in CIDP management.

A secondary finding in our case consisted in the absence of CIDP relapses during the period of mexiletine intake, even after the methotrexate drop-out (indeed, the patient withdrew by herself the methotrexate intake, since she felt better), and the abrupt CIDP relapse following mexiletine withdrawal. Altogether, these issues may configure the hypothesis of a sort of mechanism mimicking an immunomodulatory effect by part of mexiletine.^[42]^ However, this issue remains unclear since the patient did not start over again the drug. Moreover, there is scarce evidence in literature on the possible immunomodulatory effect of mexiletine, mainly limited to a drug induced hypersensitivity syndrome.^[43–46]^

In fact, it could be the case that a denervation hypersensitivity following lasting intake of mexiletine triggered the symptoms occurring after the discontinuation of the drug, rather than inducing an autoimmune reactivation.^[47–50]^ About that, experimental studies have shown that Na_v_ are over-expressed beyond the nodal-paranodal sites in the growing and sprouting axons, as well as following functional denervation. Such a remodeling of Na_v_ largely results in altered axonal and, consequently, muscle excitability.^[51–53]^ In fact, previous studies have suggested that the increased excitability of motor axons in several diseases, including amyotrophic lateral sclerosis, spinal muscular atrophy, and peripheral neuropathies, can be due to an increase in persistent sodium currents, even autoimmune-based.^[54–58]^

Last, it has to be acknowledged that peripheral neuropathy can alter the electrical properties of sensory nerves, which then leads to an imbalance between central excitatory and inhibitory signaling within inhibitory interneurons and descending control systems.^[59]^ In turn, transmission of sensory signals and disinhibition or facilitation mechanisms are altered at the level of the spinal cord dorsal horn neurons.^[59]^

Our case suggests that CIDP and MC may share some clinical and pathophysiological features, with particular regard to channelopathy, being their association not necessarily casual. Moreover, we found that some CIDP-related symptoms could benefit from Na_v_ blockers, like mexiletine. About that, mexiletine may challenge muscle and nerve excitability, regardless of its origin (ie, either a nerve hyperexcitability secondary to autoimmune-based nodal-paranodal rearrangement, or a muscle hyperexcitability following nerve damage) beyond its effectiveness on genetic muscle channelopathies. Obviously, such claim requires confirmation from clinical trials on CIDP patients, preferably without any electrophysiological evidence of muscle hyperexcitability.

## Author contributions

**Conceptualization:** Simona Portaro, Rocco Salvatore Calabrò.

**Data curation:** Simona Portaro, Fiammetta Biasini.

**Formal analysis:** Antonino Naro, Fiammetta Biasini.

**Investigation:** Simona Portaro, Fiammetta Biasini, Antonino Naro.

**Methodology:** Simona Portaro, Fiammetta Biasini, Antonino Naro.

**Supervision:** Rocco Salvatore Calabrò, Placido Bramanti.

**Validation:** Rocco Salvatore Calabrò, Antonino Naro, Placido Bramanti.

**Visualization:** Rocco Salvatore Calabrò, Placido Bramanti.

**Writing – original draft:** Simona Portaro, Antonino Naro.

**Writing – review & editing:** Simona Portaro, Antonino Naro, Rocco Salvatore Calabrò.
